# Molecular Modeling Studies on the Binding Mode of the PD-1/PD-L1 Complex Inhibitors

**DOI:** 10.3390/ijms20184654

**Published:** 2019-09-19

**Authors:** Suliman Almahmoud, Haizhen A. Zhong

**Affiliations:** 1 Department of Pharmaceutical Sciences, College of Pharmacy, University of Nebraska Medical Center, Omaha, NE 68198-6125, USA; suliman.almahmoud@unmc.edu; 2 Department of Chemistry, University of Nebraska at Omaha, Omaha, NE 68182, USA

**Keywords:** the PD-1/PD-L1 complex, protein–protein interactions, the PD-L1 protein, docking, ligand–protein interactions

## Abstract

The programmed cell death protein 1 (PD-1)/programmed cell death ligand 1 (PD-L1) is an immune checkpoint (ICP) overexpressed in various types of tumors; thus, it has been considered as an important target for cancer therapy. To determine important residues for ligand binding, we applied molecular docking studies to PD-1/PD-L1 complex inhibitors against the PD-L1 protein. Our data revealed that the residues Tyr56, Asp122, and Lys124 play critical roles in ligand binding to the PD-L1 protein and they could be used to design ligands that are active against the PD-1/PD-L1 complex. The formation of H-bonds with Arg125 of the PD-L1 protein may enhance the potency of the PD-1/PD-L1 binding.

## 1. Introduction

Immune checkpoints (ICPs) are paramount regulators of the immune system, and they can differentiate between the healthy and foreign cells and prevent activation of immune cells [1,2,3]. Cancer cells can evade immune system control by overexpressing inhibitory ICPs [4,5,6,7]. There are several co-inhibitory ICPs such as T-lymphocyte-associated protein 4 (CTLA-4), and programmed cell death protein 1 (PD-1)/programmed cell death ligand 1 (PD-L1), which inhibit T cell activation by different mechanisms [8,9,10]. Several antibodies targeting CTLA-4 or PD-1/PD-L1 have revealed encouraging clinical results [11,12]. Monoclonal antibodies (mAbs) against the PD-1 pathway show significant tumor treatment benefits, and they were considered a better option than mAbs targeting CTLA-4 [13,14,15]. Several successful mAbs targeting the PD1/PD-L1 pathway for the treatment of various tumors have been approved. These approved mAbs included nivolumab and pembrolizumab [15,16,17]. The activity of mAbs against PD1/PD-L1 checkpoints led to accelerated approval of nivolumab and pembrolizumab by regulatory bodies in 2014 [5,14].

PD-1 is a type I transmembrane immune-inhibitory protein that is expressed on activated CD4^+^ and CD8^+^ T cells, natural killer T (NKT) cells, B cells, activated monocytes, and dendritic cells (DCs) [18,19]. PD-1 and its ligands control the activity and tolerance of T cells, and immune-mediated tissue damage [19,20]. PD-1 has two ligands: PD-L1 and PD-L2; PD-L1 is expressed broadly and upregulated on activated T cells, B cells, myeloid, and dendritic cells, while PD-L2 is expressed only in activated dendritic cells and some macrophages [9,21]. In normal conditions, PD-1/PD-L1 pathways play an essential function in maintaining immune homeostasis and avoiding autoimmunity by the inhibition of T cell activation [22,23,24]. In cancer cells, the PD-1/PD-L1 interaction has a crucial role in tumor immune resistance [25,26]. The binding of the PD-1/PD-L1 complex inhibits T-lymphocyte proliferation, the release of cytokines, and induces apoptosis of T cells [27,28]. PD-L1 is overexpressed in several tumors such as lymphoma, melanoma, lung, breast cancer, glioblastoma, ovarian, kidney tumors, and bladder cancers [29,30,31,32,33,34]. Blocking of the PD-1/PD-L1 complex interaction would promote the reactivation and revival of the exhausted T cell phenotype which would normalize and stimulate the antitumor response of T cells [28]. PD-1/PD-L1 complex inhibitors represent a new type of immunotherapy drug which could afford new treatment for various kinds of cancers [35,36,37]. There are four therapeutic mAbs against PD-1/PD-L1 immune checkpoint proteins (nivolumab and pembrolizumab targeting PD-1; atezolizumab and avelumab binding to PD-L1) that have been approved by the US Food and Drug Administration (FDA). These mAbs are used to treat metastatic melanoma, non-small-cell lung cancer (NSCLC), renal cell carcinoma, head and neck squamous cell cancer (HNSCC), and bladder cancer.

However, the occurrence of immune-mediated adverse effects has been observed in some patients receiving the immune checkpoint inhibition (ICI) by mAbs. The side effects can be colitis, autoimmune hepatitis, endocrine or neurological disorders [38]. A study shows that 44.4% of patients with pre-exiting autoimmune and/or inflammatory disease (AID) experienced immune-related adverse events (irAE), whereas only 23.8% were observed with irAE in those without pre-existing AID [39]. In addition, a rapid worsening of the disease upon treatment with ICIs was observed in approximately 5% of patients, a phenomenon called hyper-progressive disease (HPD) and it seems to be due to inhibition of both PD-1 and PD-L1 [40,41].

Therefore, a small molecule inhibitor that binds to PD-L1 only would spare the function of PD-1 and may be an alternative therapeutic approach that may minimize the immune-related adverse effects. Unfortunately, the development of small molecules targeting the PD-1/PD-L1 axis lags far behind the mAb development targeting the same pathway. This is due, in part, to insufficient structural information of the PD-1/PD-L1 complex with small molecule inhibitors. The crystal structure of the fully human PD-1/PD-L1 complex (Protein Data Bank Identification Code, PDB ID: 4ZQK) that was determined in this crystal structure suggests that there are several binding sites on the PD-1 and PD-L1 that can be targeted to develop small molecule inhibitors [42]. The crystal structures of PD-L1 complexed with atezolizumab (PDB ID: 3X8L) and durvalumab (PDB ID: 3X8M) were not available until 2017, and they defined the binding site and important residues of the PD-L1 interacting with the mAbs [43]. Recently, some new small molecules were identified as PD1/PD-L1 pathway inhibitors with significant inhibitory effects at subnanomolar concentrations [44,45]. Besides, the crystal structures of PD-L1 complexed with small molecule inhibitors have been resolved showing that small molecules bind to PD-L1 instead of PD-1, and they inhibit the PD-1/PD-L1 interaction by inducing PD-L1 dimerization through the PD-1 interacting surface site. The binding of small molecules to PD-L1 led to disassociation of the PD-1/PD-L1 complex [46,47,48].

**Figure 1:**
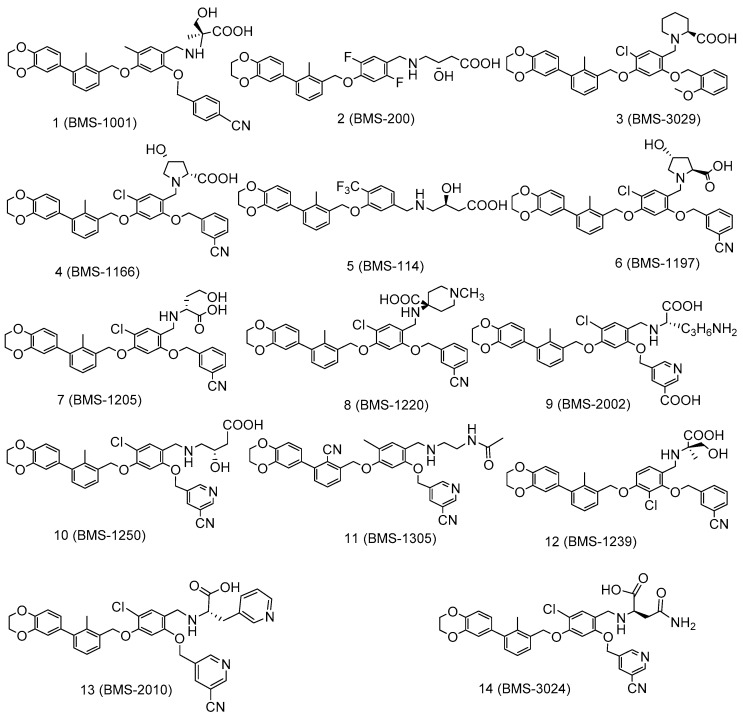
Structures of the programmed cell death protein 1 (PD-1)/programmed cell death ligand 1 (PD-L1) complex inhibitors (1–14) used in docking studies.

**Figure 2:**
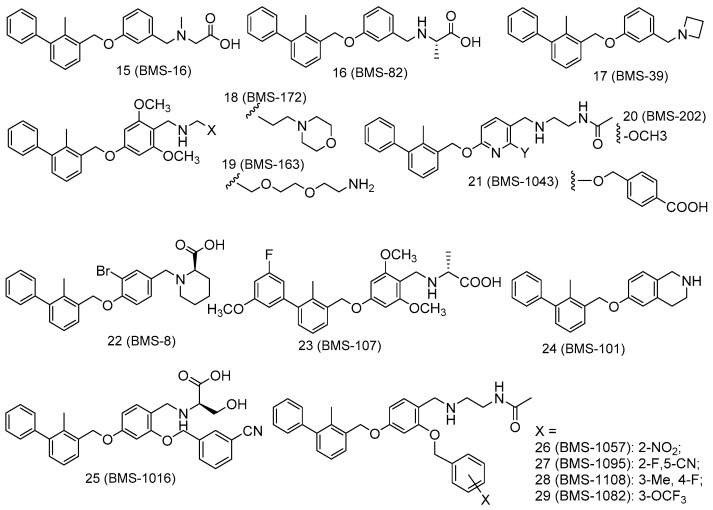
Structures of the PD-1/PD-L1 complex inhibitors (15–29) used in docking studies.

Here, we report the structural basis for PD-L1 interactions with the reported Bristol-Myers Squibb (BMS) inhibitors (Figure 1 and Figure 2). The docking scores of BMS inhibitors showed that the docking scores in our study are very close to those experimentally observed biological activities. The binding modes and protein–ligand interaction studies of the PD-L1/BMS inhibitors offer significant structural insight for the design and development of future new and selective inhibitors for the PD-1/PD-L1 complex. We identified residues Tyr56, Asp122, and Lys124 of PD-L1 as important binding residues for small molecule design as they can form H-bond interactions. Tyr56 and Asp122 appear to be the two most vital residues for interaction with PD1/PD-L1 complex inhibitors.

## 2. Results and Discussion

### 2.1. Docking Scores and Validation

To identify amino acids that are essential for small molecule binding, we carried out docking studies of 29 experimentally verified inhibitors of the PD-1/PD-L1 complex (Figure 1 and Figure 2). These 29 ligands were docked to two different PD-L1 model proteins (PDBIDs: 5NIU [48] and 5N2F [47]). The reason for using two crystal structures for docking studies was to determine the consistency of docking findings being independent of a given protein structure. Note that 5NIU is a tetramer with two identical bound ligands and thus we randomly chose chains C and D and their bound ligand for docking studies. The PD-L1/ligand docking scores for these two models are listed in Table S1. The docking scores show that the glide performance was well within the predicted range of the binding affinity (ΔG_PRED_) of PD-1/PD-L1 complex inhibitors. The comparisons of predicted docking scores to the experimental free energy of bindings, converted from the IC_50_s, show that the docking scores of both models 5NIU and 5N2F are in good agreement with the experimentally observed data, with mean errors of 1.07 and 0.91 kcal/mol, and the root-mean-square errors of 1.66 and 1.51 kcal/mol for model proteins 5NIU and 5N2F, respectively. The low standard deviations of 1.29 and 1.22 for model proteins 5NIU and 5N2F, respectively, further confirm the validity of the glide docking method and confirm the consistency of the docking studies in our PD-L1 system. The more negative the docking score, the more favorable the interaction of the complex. To determine the protonation state of amino groups in the compounds outlined in Figure 1 and Figure 2, we carried out computational pKa calculations using EPik program. Table S2 shows that all compounds show a pKa around 8 except BMS-1220 (**8**), which has a high pKa of 10. This suggests that the nitrogen atom on most ligands should not be protonated and thus remains neutral, whereas BMS-1220 (**8**) was protonated.

In addition to binding affinity, ligand binding can also be evaluated by binding free energy (ΔG). Herein we use a knowledge-based moveable-type (MT)-based approach [49] to estimate the absolute free energy of the binding of all 29 ligands using the docked poses identified in the docking study for two model proteins. The MT-based free energy calculation has been successfully applied to engineering cellular retinoic acid binding protein II [50].

**Figure 3:**
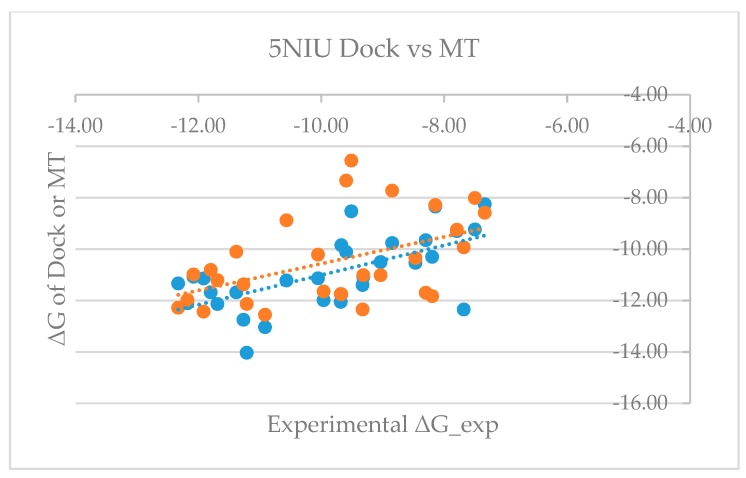
Plot of experimental binding free energy (ΔG) versus glide dock-based and moveable-type (MT)-based ΔG for the 5NIU model (blue: 5NIU_Dock, orange: 5NIU_MT).

**Figure 4:**
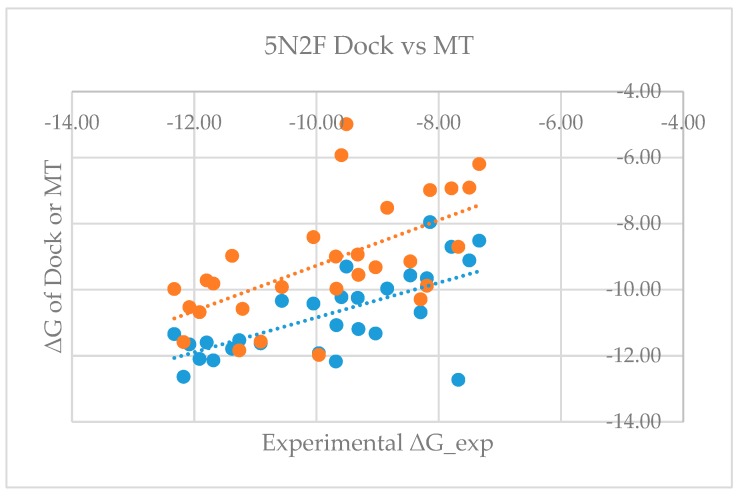
Plot of experimental ΔG versus glide dock-based and MT-based ΔG for the 5N2F model (blue: 5N2F_Dock, orange: 5N2F_MT).

Table S3 shows that the mean errors of predicted free energy of binding from the corresponding experimental values are 0.64 and −0.68 kcal/mol, with standard deviations of 1.68 and 1.54, and RMSEs of 1.77 and 1.66 for model proteins 5NIU and 5N2F, respectively. The good agreement between the predicted and the experimentally observed values not only proves the validity of the MT-based free energy calculation method, but also further validates the glide-dock program because the generated docked poses can be used to accurately predict the binding affinity. Figure 3 and Figure 4 show the dot plots between experimental ΔG versus glide dock-based (blue) and MT-based ΔG (orange) and suggest that the docking appeared to systematically overestimate the binding free energy and thus appears to have a relatively larger mean error. Tables S1 and S3 revealed that mean errors for the docking of the 5NIU and 5N2F model proteins were 1.07 and 0.91, whereas those errors from the MT method were 0.64 and −0.68, respectively.

**Figure 5:**
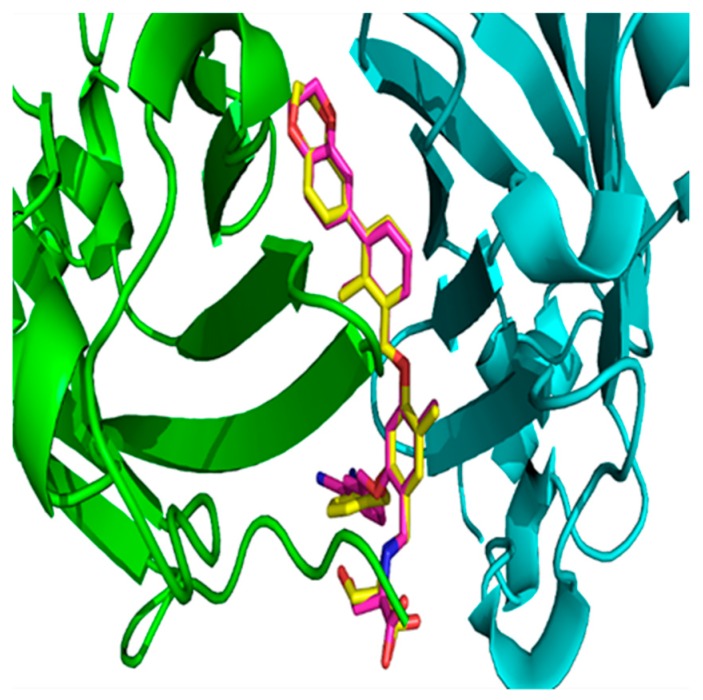
The superposition of the glide-docked generated pose and its native conformation in 5NIU for ligand BMS-1001 (**1**, 5NIU)**.** The native confirmation is in yellow color, and the docked pose is in magenta color. Chain D is colored with a green secondary structure, whereas Chain C is in cyan color.

There are other methods to validate docking methods [51,52]. The pose selection is a standard method used whereby docking software is used to dock a ligand with a known conformation and orientation, typically from a co-crystal structure, into the binding site. The docking software is considered dependable when it is able to generate a pose that is very close to the native conformation in the crystal structure, i.e., the root-mean-square deviation (RMSD) value between the docked pose and the native conformation is low (less than 1.5 or 2 Å depending on ligand size) [53]. The superposition of the glide-generated docked pose, and the native conformation in the co-crystal structure (PDB ID: 5NIU) for compound **1** (Figure 5) showed that the RMSD between these two poses is 1.04 Å. The RMSD value between the docked pose and the native structure in 5N2F was 0.79 Å (Figure S1). Therefore, the low RMSD values from both models confirmed that the glide dock is able successfully to find the native poses in crystal structures and can be reliably used to define the binding conformations of other ligands.

**Figure 6:**
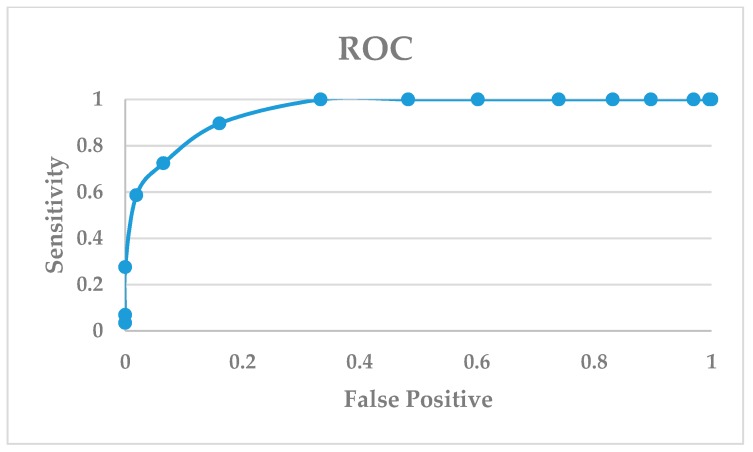
Docking enrichment plot of false positive vs. sensitivity in the 5NIU model.

Another validation method to evaluate a dock program is the enrichment factor (EF) [54,55,56]. The EF measures the concentration of the active and known inhibitors in a specific subset divided by the concentration of the active and known inhibitors in the database. The EF is a general measurement of the efficiency of a docking program: The higher the EF, the more accurate the docking program. The EF can validate a docking program if it can satisfactorily identify the active PD-L1 inhibitors from a database of drug-like molecules. To do so, we docked a library of 29 PD-L1/PD-1 complex inhibitors along with 261 drug-like compounds against the crystal structure of PD-L1. The docking of 290 compounds against the crystal structure of the PD-L1 (PDB: 5NIU) model resulted in an EF score of 8.62, which is calculated by [EF = (25/29)/(29/290)]. Another way to evaluate the sensitivity of a docking program is to use a receiver operator curve (ROC) in which the frequency of a false positive is plotted against the sensitivity. The false positives refer to drug-like molecules being top-ranked from the docking output. The docking output of 261 drug-like molecules was listed in Table S4. Figure 6 shows that the glide dock program is quite sensitive in identifying true positives (i.e., active compounds) in our study case.

### 2.2. Binding Interactions of PD-L1/inhibitors

After the validity of the glide dock method was confirmed by the aforementioned methods, we can confidently use the docked poses to identify PD-L1/inhibitor interactions. To design new molecules with the desired potency, it is important for the designed molecules to maintain the proper interactions with essential residues in the binding pocket. Thus, it is very important to identify critical binding residues for effective PD-L1 binding.

**Figure 7:**
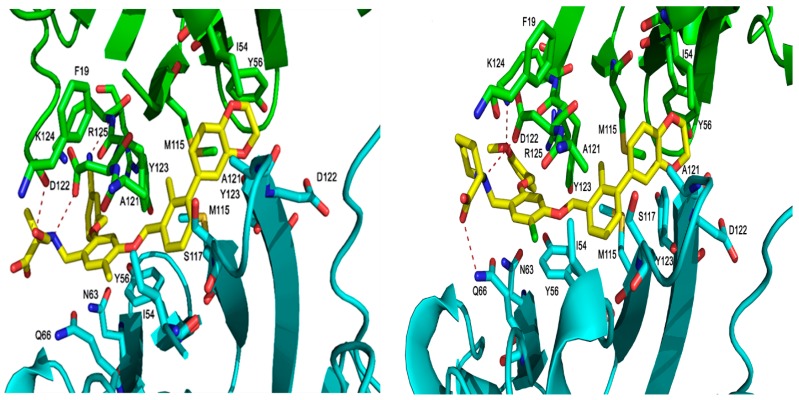
The binding orientation of compound **1** (left), and compound **3** (right) in the PD-L1 protein of 5NIU. The H-bond is depicted with a dark dotted line. Chain D is colored with a green secondary structure and atoms, whereas Chain C is in cyan color.

The PD-1/PD-L1 complex inhibitors bind to PD-L1 through the PD-1 interacting surface site inducing PD-L1 dimerization and disassociation of a PD-1/PD-L1 complex. In the crystal structure of 5NIU (PD-L1 dimer/BMS-1001 (**1**)), the 2, 3-dihydro-1, 4-benzodioxine fragment creates π–π stacking interaction with Tyr56, and the (2R)-2- amino-3-hydroxypropanoic acid moiety formed H-bonds with the carbonyl of Asp122, Tyr123, Lys124, and the main chain carbonyl oxygen of Phe19 (Figure 7). Besides, the 3-cyanobenzyl moiety partly creates a π–π stacking interaction with Tyr123, and forms hydrogen bonds with Arg125. The absence of the 3-hydroxy group in inhibitor **3** means it is unable to H-bond with Arg125 and Phe19 (Figure 7), resulting in a much weaker interaction. The IC_50_s for compounds **1** and **3** are 2.25 and 2350 nM, respectively. Our MT-based binding free energy calculations predicted ΔGs of −10.81 and −9.94 kcal/mol, respectively, showing a weaker binding in compound **3**.

The PD-1/PD-L1 complex inhibitors were run *in silico* docking using the glide docking method to identify the binding mechanisms of these compounds. The protein/ligand interactions might vary due to the different structural nature of each ligand. To identify residues that are responsible for most ligand binding, we enumerated residues that form H-bonds, or electrostatic interactions or π–π stack interactions with 29 inhibitors. Table 1 shows that residues Tyr56 and Asp122 are the two most important residues for ligand binding.

**Figure 8:**
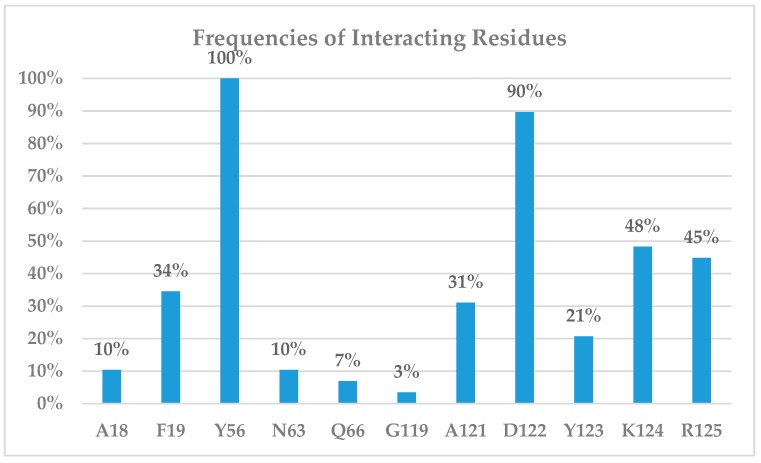
Interacting residues of PD-L1 with all 29 different inhibitors in the 5NIU model.

To evaluate the relative importance of active site residues in ligand binding, we enumerate all binding residues for all 29 ligands. Figure 8 shows that Tyr56 interacts with all 29 inhibitors and Asp122 forms H-bonds with 90% of the studied compounds. In addition, Lys124, Arg125, and Phe19 are important residues for ligand binding as they appear between 30 and 50% ligand binding. The positively charged nature of Lys124 and Arg125 suggests that a negatively charged carboxylate moiety is likely expected in PD-L1 inhibitors. Please note that, to avoid over-exaggeration of the contributions of binding residues, if a residue appears in both chain C and chain D, it is only counted as one. For instance, Tyr56 of chains C and D provides π–π stack interactions with the aromatic rings of ligands but was only counted once for each entry for compounds **2**, **5**, **6**, **7**, and so on. The potency of inhibitors toward the PD-1/PD-L1 complex might be attributed to their ability to interact with Arg125. The majority of potent PD-1/PD-L1 complex inhibitors with IC_50_ of 100 nM or better tend to show interactions with Arg125, as observed in the potent compound. We also investigated the protein–ligand interactions for the 5N2F model (Table S5), and the frequencies of interacting residues are reported in Figure S2. Table S5 and Figure S2 show that Tyr56 and Asp122 are the most important residues for ligand binding. Like what is observed in the 5NIU model, Lys124 is likely to be important in ligand binding. However, the 5N2F model added two new residues for ligand binding: Ala18 and Thr20, with Phe19 showing reduced significance.

**Figure 9:**
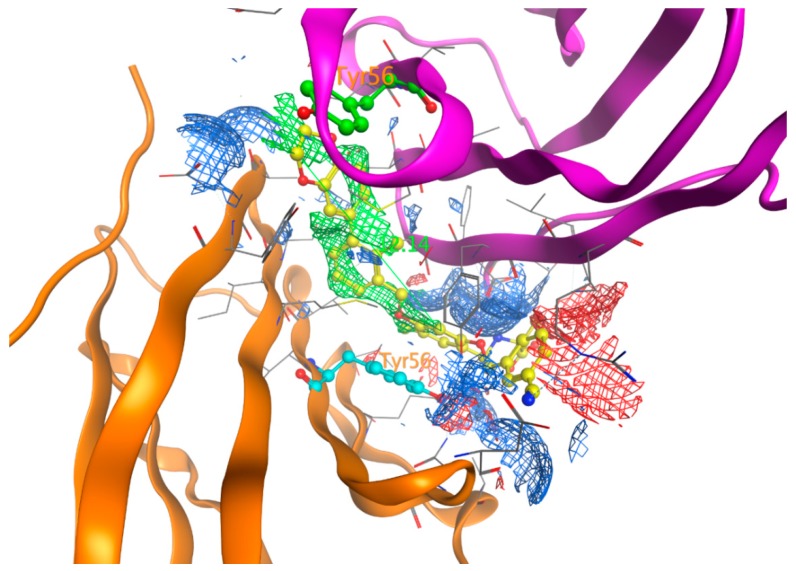
Electrostatic surface of the binding pockets of the PD-L1 with BMS-1001 (**1**, 5NIU). The hydrophobic region is depicted as green; H-bond acceptor, red; and H-bond donor, blue. Chain D is colored with a magenta secondary structure, whereas Chain C is in orange color. Tyr56 of chain C is highlighted in cyan and Tyr56 of Chain D in green. The distance between the two aromatic rings interacting with Tyr56 of chains C and D is 12.14 Å.

Though ligands tend to bind to the interface of dimer Chains C and D, they prefer binding to one chain over the other; in this case, they show closer interactions with chain D residues as evidenced by Table 1 and Figure 7. The most frequent residue from chain C is Tyr56, which, along with the same residue from chain D, forms two π–π stack interactions with two aromatic rings of inhibitors. This suggests that there should be two aromatic rings separated by 12 Å for PD-L1 inhibitors to interact with Tyr56 from both chains (Figure 9).

The electrostatic map of PD-L1/ BMS-1001 (**1**, 5NIU, Figure 9) further confirms that Chain D plays a significant role in ligand binding, whereas the role of chain C is much less because the latter has fewer interactions with the PD-L1 inhibitors. The phenol group of Tyr56 is exposed to the binding site generating π–π stacking with the inhibitors. The carboxyl group of Asp122 was positioned toward the ligand binding, with a high concentration of negative charge from the carboxylate group, and served as an H-bond acceptor with the compound **1**. This high concentration of negative charge is visible as red regions in the plot of electrostatic potential (Figure 9). The PD-L1 binding sites have a high concentration of positive charge featuring Lys124 and Arg125 that bind the compound; this high concentration of positive charge is visible as blue regions in the electrostatic potential map (Figure 9). This observation is supported by the high frequency of Lys124 and Arg125 in the protein–ligand interaction map (Figure 8). Therefore, future PD-L1 inhibitor design should consider the residues Tyr56, Asp122, and Lys124 along with two aromatic rings. The importance of residue Tyr56 has already been observed and reported [46,47,48]. The finding of Asp122, and Lys124, and two essential aromatic rings may provide helpful guidelines for future PD-L1 inhibitor design.

## 3. Computational Methods

### 3.1. Preparation of Protein Structures

The X-ray crystal structures of the human wild type PD-L1/BMS-1001 (1, PDBID: 5NIU) [48] and the structure of PD-L1/BMS-200 (2, PDBID: 5N2F) [47] were downloaded from the Research Collaboratory for Structural Bioinformatics (RCSB) protein data bank (available online: https://www.rcsb.org/structure/). No missing residues were observed in these two crystal structures except a few residues with missing parts of side chains. The missing side chains were regenerated during the protein preparation step in MOE to correct the missing side chains, to optimize the hydrogen bonding network, to allow protonation to be assigned to charged residues, and to allow the flipping of the side chains of Asn, Gln, and His in MOE to maximize H-bond interactions [57]. Due to their not serving as a bridge between protein and ligands, 1,2-ethanediol and the water molecules were deleted in the protein preparation step. Subsequently, the X-ray crystal structure was subjected to energy minimization using the Amber14:EHT forcefield [58] in MOE, followed by protein preparation using the Protein Preparation Wizard in the Schrödinger software [59] to allow the flipping of the side chains of the residues of Asn and Gln to maximize H-bond interactions. Then, they were subjected to energy minimization with a protein backbone by using the OPLS3 forcefield in the MacroModel module in the Maestro before the docking procedure.

### 3.2. Preparation of Ligands and Molecular Docking

We built 29 PD-1/PD-L1 complex inhibitors (Figure 1 and Figure 2) from different sources [44,45] based on the crystal structure of 1 in 5NIU as a template using the MOE build panel and all model molecules were subjected to energy minimization using the MMFF94× forcefield partial charges in MOE [60]. The minimized ligands were imported to Maestro in Schrödinger software suite for proper treatment before docking. All inhibitors were minimized by the MacroModel module by using the OPLS2005 forcefield [59]. The pKa calculations of ligands were prepared by using the Epik program in the Schrödinger software [59]. The Epik calculations calculated the pKa values of all nitrogen atoms in these ligands and if an amine has a pKa greater than 10, that amino group will be protonated. Otherwise, it would be treated as neutral. In our case, only compound BMS-1220 (**8**) has a pKa of 10.73, which is greater than 10 and thus should be protonated. The pKa values of nitrogen atoms of all 29 inhibitors are listed in Supplementary Information Table S1.

To calculate the enrichment factor in order to evaluate the effectiveness of the docking program, we downloaded a database of 260,071 ligands from the National Cancer Institute (NCI) [61] and this database was converted to 3D structures, ionic components were removed, the database was further filtered with the Lipinski’s rule of five [62], and then 261 molecules were randomly selected from this database to assess the enrichment factor and validate our docking study. The selected 261 molecules were subjected to energy minimization using the MOE and MacroModel programs. The combined database of 29 inhibitors along with 261 randomly drug-like molecules were docked to the PD-L1 binding pockets of model proteins 5NIU and 5N2F.

The compounds were then subjected to energy minimization using the MacroModel module in Maestro [59]. We used Glide Dock in Maestro 11.6 for the docking of inhibitors to the PD-L1 proteins [59]. Consequently, we generated a grid file for the crystal structures of 5NIU and 5N2F using the glide grid generation protocol with the bound ligands as centroids of the protein binding pocket [59]. All 29 inhibitors were docked into the grid file, and we later ran docking for the 261 NCI drug-like molecules using the same grid files. During the docking process, the scaling factor for receptor Van der Waals for the nonpolar atoms was set to 0.8 to allow for some flexibility of the receptor. Besides, all other parameters were used as defaults and the docking procedure was established [63]. The binding affinity of the PD-L1/ligand complexes was expressed in terms of docking scores. The output docking scores were defined as ΔG_PRED_. The output ΔG_PRED_ was then related to the experimental ΔG_EXP_, which calculated from the experimental IC_50_ (nM) using the following equation:

$${\Delta G}_{EXP}\left(kcal/mol\right)=\;RT\ln\;\left(IC_{50\;}\left(nM\right)\times 10^{-9}\right)/1000\;\;{\;}_{\;}$$

Furthermore, we created an electrostatic map for the binding site of PD-L1 to estimate the electrostatically favored locations of H-bond donors, H-bond acceptors, and hydrophobic interactions. The electrostatic map was made using the MOE program [57]. The receiver operator curve (ROC) was plotted based on the frequencies of false positives and that of true active compounds (sensitivity) and was calculated based on the docking scores of active compounds and drug-like molecules.

### 3.3. Binding Free Energy Calculations Using the Moveable-Type (MT)-Based Approach

After 29 inhibitors were docked to the 5NIU protein, the model protein was saved in PDB format and the 29 ligands were each saved separately in mol2 file format. The saved protein and ligand files were fed as input files for the movable-type (MT) free energy calculation method, an in-house program developed by Prof. Kenneth Merz Jr. at Michigan State University and which was generously given to us for complimentary use. The output data of the MT-based method is the absolute free energy of binding; these data were used to compare with those experimentally observed values and are listed in Table S3. The reliability of this method has been in previous publications. [49,50].

## 4. Conclusions

The prevalence of the PD-1/PD-L1 complex in several human cancer cells has made it an attractive target for anticancer drug discovery. The positive results of mAb targeting the PD-1/PD-L1 complex in cancer treatment are boosting and inspiring the design and development of small molecules targeting this pathway. Our studies on the protein/ligand dockings and structural analysis on the docked complexes have suggested that the glide dock approach and the MT-based free energy calculation method are very dependable in terms of their predictability, with low error compared to those observed values. The low RMS deviations of the docked pose to the native conformation and the very good enrichment factor further confirm the effectiveness of the Glide Dock program. The analyses of the protein/ligand interactions reveal that PD-L1 residues Tyr56, Asp122, and Lys124 may be very important for inhibitor design and that two aromatic rings may be expected in new PD-L1 inhibitors. From a drug design point of view, Phe19 and Asp122 can interact with the amino moiety of ligands and Lys124 and Arg125 provide interactions the carboxylate groups of ligands whereas Tyr56 from chains C and D, forming π–π interactions with two different aromatic rings as shown in compound **9** (Figure S3). All these observations will be very useful in the design, development, and discovery of the next generation of potent PD-1/PD-L1 complex inhibitors.

## Figures and Tables

**Table 1:** The ligand–protein interactions between the PD-1/PD-L1 complex inhibitors and the PD-L1 protein of 5NIU.

Title	IC50 (nM)	Chain C	Chain D
BMS-1001(1, 5NIU)	2.25		Tyr56, Asp122, Lys124, Arg125, Phe19
BMS-200 (2, 5N2F)	80	Tyr56	Tyr56, Ala121, Asp122
BMS-3029 (3)	2350	Tyr56, Gln66	Tyr56, Asp122, Tyr123, Lys124
BMS-1166 (4, 5NIX)	1.4		Tyr56, Asp122, Arg125
BMS-114 (5)	43	Tyr56	Tyr56, Asp122, Arg125
BMS-1197 (6)	1.85	Tyr56	Tyr56, Asp122, Lys124, Arg125, Phe19
BMS-1205 (7)	2.71	Tyr56, Gln66	Tyr56, Asp122, Lys124, Arg125
BMS-1220 (8)	6.07		Tyr56, Asp122, Lys124, Arg125
BMS-2002 (9)	10	Tyr56	Tyr56, Ala121, Asp122, Tyr123, Lys124, Arg125, Phe19
BMS-1250 (10)	1.19	Tyr56	Tyr56, Ala121, Asp122, Arg125, Ala18, Phe19
BMS-1305 (11)	0.92	Tyr56	Tyr56, Asp122, Tyr123, Arg125
BMS-1239 (12)	148.9		Tyr56, Asp122, Lys124
BMS-2010 (13)	50		Tyr56, Asp122, Lys124, Arg125, Ala18
BMS-3024 (14)	5.54	Gln66	Tyr56, Asp122, Arg125, Phe19
BMS-16 (15)	1945	Tyr56, Asn63	Tyr56, Asp122
BMS-82 (16)	3186		Tyr56, Ala121, Phe19, Ala18
BMS-39 (17)	4184	Tyr56	Tyr56, Asp122
BMS-172 (18)	107	Tyr56	Tyr56, Ala121, Asp122, Tyr123
BMS-163 (19)	93	Tyr56	Tyr56, Gly119, Ala121, Asp122, Tyr123
BMS-202 (20, 5J89)	18	Tyr56	Tyr56, Ala121, Asp122
BMS-1043 (21)	239.2		Tyr56, Ala121, Asp122, Tyr123, Lys124, Phe19
BMS-8 (22, 5J8O)	146	Asn63	Tyr56, Lys124
BMS-107 (23)	329		Tyr56, Asp122, Lys124
BMS-101 (24)	1076	Gln66	Tyr56
BMS-1016 (25)	4.55	Tyr56	Tyr56, Asp122, Arg125
BMS-1057 (26)	985.8	Tyr56	Tyr56, Asp122, Lys124, Phe19
BMS-1095 (27)	81.25	Tyr56	Tyr56, Ala121, Asp122, Lys124, Arg125, Phe19
BMS-1108 (28)	624.2	Asn63	Tyr56, Asp122
BMS-1082 (29)	828.4		Tyr56, Ala121, Asp122, Lys124, Phe19

## Supplementary Materials

Supplementary materials can be found at https://www.mdpi.com/1422-0067/20/18/4654/s1.
