# Supplementary material for: Molecular Modeling Studies on the Binding Mode of the PD-1/PD-L1 Complex Inhibitors
Source: Int J Mol Sci. 2019 Sep 19;20(18):4654. doi: 10.3390/ijms20184654 (PMC6770281; doi:10.3390/ijms20184654)
Supplement: Supplementary file 1 [file ijms-20-04654-s001.pdf]

Molecular Modeling Studies on the Binding Mode of the PD-1/PD-L1 Complex Inhibitors

Suliman Almahmoud [1] and Haizhen A. Zhong [2,*]

[1] Department of Pharmaceutical Sciences, College of Pharmacy, University of Nebraska Medical Center, Omaha, Nebraska 68198-6125, USA
[2] Department of Chemistry, University of Nebraska at Omaha, Omaha, Nebraska 68182, USA
* Correspondence: hzhong@unomaha.edu; Tel.: +1-402-554-3145

Table S1: The glide docking scores (unit, kcal/mol) of **29** ligands against PD-L1 proteins (5NIU and 5N2F).

Title	IC50 (nM)	ΔG(exp)	5NIU_Dock	ΔΔG (5NIU)	5N2F_Dock	ΔΔG (5N2F)
**BMS-1001(1, 5NIU)**	2.25	-11.80	-11.69	-0.11	-11.60	-0.20
**BMS-200 (2, 5N2F)**	80	-9.68	-12.06	2.38	-12.18	2.50
**BMS-3029 (3)**	2350	-7.68	-12.35	4.68	-12.73	5.05
**BMS-1166 (4, 5NIX)**	1.4	-12.08	-11.08	-1.00	-11.66	-0.42
**BMS-114 (5)**	43	-10.05	-11.14	1.09	-10.42	0.37
**BMS-1197 (6)**	1.85	-11.91	-11.15	-0.76	-12.10	0.18
**BMS-1205 (7)**	2.71	-11.69	-12.14	0.46	-12.14	0.45
**BMS-1220 (8)**	6.07	-11.21	-14.04	2.83	-10.58	-0.63
**BMS-2002 (9)**	10	-10.91	-13.04	2.13	-11.63	0.71
**BMS-1250 (10)**	1.19	-12.17	-12.11	-0.06	-12.64	0.46
**BMS-1305 (11)**	0.92	-12.33	-11.35	-0.98	-11.34	-0.99
**BMS-1239 (12)**	148.9	-9.31	-11.10	1.79	-11.19	1.88
**BMS-2010 (13)**	50	-9.96	-12.00	2.04	-11.93	1.97
**BMS-3024 (14)**	5.54	-11.26	-12.75	1.49	-11.53	0.27
**BMS-16 (15)**	1945	-7.79	-9.31	1.52	-8.70	0.91
**BMS-82 (16)**	3186	-7.50	-9.24	1.74	-9.12	1.62
**BMS-39 (17)**	4184	-7.34	-8.26	0.92	-8.52	1.18
**BMS-172 (18)**	107	-9.51	-8.54	-0.97	-9.30	-0.21
**BMS-163 (19)**	93	-9.59	-10.12	0.53	-10.23	0.63
**BMS-202 (20, 5J89)**	18	-10.56	-11.22	0.66	-10.34	-0.23
**BMS-1043 (21)**	239.2	-9.03	-10.51	1.48	-11.33	2.29
**BMS-8 (22, 5J8O)**	146	-9.32	-11.40	2.08	-10.25	0.92
**BMS-107 (23)**	329	-8.84	-9.77	0.93	-9.97	1.12
**BMS-101 (24)**	1076	-8.14	-8.35	0.21	-7.96	-0.19
**BMS-1016 (25)**	4.55	-11.38	-11.69	0.31	-11.79	0.41
**BMS-1057 (26)**	985.8	-8.19	-10.31	2.12	-9.66	1.46
**BMS-1095 (27)**	81.25	-9.67	-9.86	0.18	-11.07	1.40
**BMS-1108 (28)**	624.2	-8.46	-10.55	2.08	-9.57	1.11
**BMS-1082 (29)**	828.4	-8.30	-9.66	1.37	-10.68	2.39
**Mean Error**				1.07		0.91
**STDev**				1.29		1.22
**RMSE**				1.66		1.51

Table S2. The pKa values of ligands as predicted by the EPik program.

		**Title**	**pK_a**
**BMS-1001(1, 5NIU)**	8.28	**BMS-82 (16)**	8.63
**BMS-200 (2, 5N2F)**	8.76	**BMS-39 (17)**	8.79
**BMS-3029 (3)**	8.74	**BMS-172 (18)**	8.77
**BMS-1166 (4, 5NIX)**	8.72	**BMS-163 (19)**	8.2
**BMS-114 (5)**	8.79	**BMS-202 (20, 5J89)**	7.74
**BMS-1197 (6)**	8.72	**BMS-1043 (21)**	8.08
**BMS-1205 (7)**	8.48	**BMS-8 (22, 5J8O)**	8.71
**BMS-1220 (8)**	10.73	**BMS-107 (23)**	8.72
**BMS-2002 (9)**	8.19	**BMS-101 (24)**	8.76
**BMS-1250 (10)**	8.87	**BMS-1016 (25)**	8.27
**BMS-1305 (11)**	8.11	**BMS-1057 (26)**	8.08
**BMS-1239 (12)**	8.48	**BMS-1095 (27)**	8.08
**BMS-2010 (13)**	8.31	**BMS-1108 (28)**	8.08
**BMS-3024 (14)**	8.34	**BMS-1082 (29)**	8.08
**BMS-16 (15)**	8.23		

Table S3: The Moveable-Type-based binding free energy (unit, kcal/mol) of **29** ligands against PD-L1 proteins (5NIU and 5N2F), using docked poses from the Glide dock program.

Title	IC50 (nM)	ΔG(exp)	MT_5NIU	ΔΔG (5NIU)	MT_5N2F	ΔΔG (5N2F)
**BMS-1001(1, 5NIU)**	2.25	-11.80	-10.81	-0.98	-9.72	-2.08
**BMS-200 (2, 5N2F)**	80	-9.68	-11.75	2.07	-9.00	-0.68
**BMS-3029 (3)**	2350	-7.68	-9.94	2.26	-8.70	1.02
**BMS-1166 (4, 5NIX)**	1.4	-12.08	-10.99	-1.09	-10.53	-1.55
**BMS-114 (5)**	43	-10.05	-10.22	0.17	-8.41	-1.64
**BMS-1197 (6)**	1.85	-11.91	-12.44	0.53	-10.68	-1.23
**BMS-1205 (7)**	2.71	-11.69	-11.22	-0.47	-9.82	-1.87
**BMS-1220 (8)**	6.07	-11.21	-12.14	0.93	-10.58	-0.63
**BMS-2002 (9)**	10	-10.91	-12.56	1.65	-11.57	0.66
**BMS-1250 (10)**	1.19	-12.17	-11.99	-0.19	-11.58	-0.59
**BMS-1305 (11)**	0.92	-12.33	-12.29	-0.04	-9.98	-2.35
**BMS-1239 (12)**	148.9	-9.31	-11.02	1.71	-9.55	0.24
**BMS-2010 (13)**	50	-9.96	-11.65	1.69	-11.97	2.01
**BMS-3024 (14)**	5.54	-11.26	-11.37	0.11	-11.84	0.57
**BMS-16 (15)**	1945	-7.79	-9.25	1.46	-6.93	-0.86
**BMS-82 (16)**	3186	-7.50	-8.02	0.52	-6.91	-0.59
**BMS-39 (17)**	4184	-7.34	-8.59	1.26	-6.19	-1.14
**BMS-172 (18)**	107	-9.51	-6.57	-2.94	-4.99	-4.52
**BMS-163 (19)**	93	-9.59	-7.34	-2.25	-5.93	-3.67
**BMS-202 (20, 5J89)**	18	-10.56	-8.89	-1.68	-9.92	-0.64
**BMS-1043 (21)**	239.2	-9.03	-11.02	1.99	-9.32	0.29
**BMS-8 (22, 5J8O)**	146	-9.32	-12.35	3.03	-8.94	-0.39
**BMS-107 (23)**	329	-8.84	-7.73	-1.11	-7.52	-1.32
**BMS-101 (24)**	1076	-8.14	-8.29	0.15	-6.98	-1.16
**BMS-1016 (25)**	4.55	-11.38	-10.11	-1.27	-8.98	-2.40
**BMS-1057 (26)**	985.8	-8.19	-11.84	3.64	-9.88	1.69
**BMS-1095 (27)**	81.25	-9.67	-11.76	2.09	-9.97	0.30
**BMS-1108 (28)**	624.2	-8.46	-10.35	1.89	-9.15	0.68
**BMS-1082 (29)**	828.4	-8.30	-11.71	3.41	-10.29	2.00
**MAE**				0.64		-0.68
**STDev**				1.68		1.54
**RMSE**				1.77		1.66

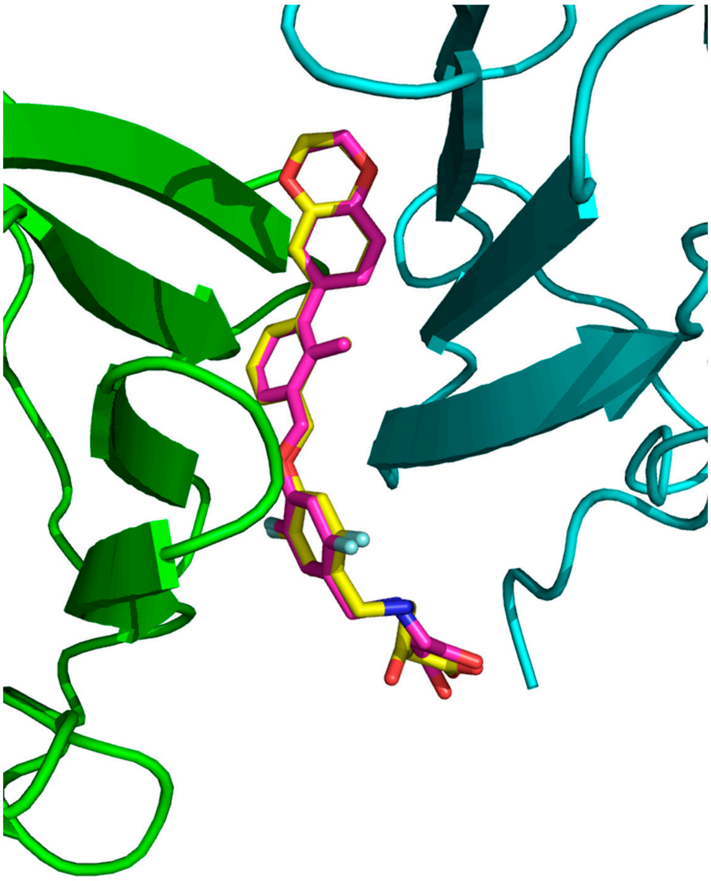

Figure S1: The superposition of the glide-docked generated pose and its native conformation in 5N2F for ligand BMS-200(**2**, 5N2F)**.** The native confirmation is in yellow color, and the docked pose is in magenta color. Chain D is colored with green secondary structure and whereas Chain C in cyan color.

Table S4. Glide docking scores of 261 drug-like molecules obtained from an NCI database against the PD-L1 protein (5NIU).

NCI ID	Docking Scores	NCI ID	Docking Scores	NCI ID	Docking Scores	NCI ID	Docking Scores
**66**	-1.90	113321	-9.27	221630	-8.50	512107	-7.43
**1185**	-4.12	113554	-4.04	230200	-4.66	521770	-4.01
**1229**	-7.67	114380	-9.36	230218	-5.82	602688	-3.37
**6594**	-5.34	116101	-4.02	230339	-9.58	610172	-5.77
**8007**	-10.33	116505	-8.66	231593	-5.12	610529	-8.11
**8796**	-1.82	117598	-1.88	231919	-7.66	614534	-6.53
**21357**	-7.76	119770	-7.94	237021	-3.00	615401	-2.25
**22645**	-9.00	121111	-8.85	247507	-6.99	615839	-6.99
**24026**	-8.48	121426	-5.37	249991	-5.10	618629	-2.94
**25686**	-8.88	124018	-11.98	251798	-3.25	623064	-6.11
**26870**	-5.42	125048	-9.71	259678	-6.09	626099	-6.64
**26873**	-8.85	125658	-7.02	270462	-2.43	627595	-2.62
**30850**	-3.96	126152	-9.15	275635	-10.75	627988	-4.36
**31781**	-5.61	132516	-5.20	277563	-5.73	628670	-4.17
**34575**	-4.94	134098	-6.36	281758	-8.79	628992	-2.58
**35739**	-8.18	134953	-3.51	285667	-9.00	631580	-7.77
**35812**	-9.64	135757	-5.57	288368	-3.94	631845	-9.46
**37358**	-4.86	135906	-3.99	289517	-11.92	631849	-5.17
**38206**	-5.51	142487	-5.70	291629	-7.21	632252	-9.97
**38224**	-6.09	142549	-10.46	294195	-9.21	634620	-8.08
**40436**	-10.72	144471	-3.97	295720	-9.02	639645	-5.23
**45231**	-1.89	147670	-4.69	295740	-7.00	640635	-4.32
**47705**	-9.63	149079	-6.85	297359	-9.30	641095	-11.32
**47711**	-7.88	149802	-10.12	297621	-5.39	641615	-3.96
**48191**	-5.54	154683	-3.07	300505	-8.40	642066	-5.29
**49810**	-4.92	155336	-3.17	302647	-7.02	642740	-6.30
**50410**	-8.57	157443	-8.57	303242	-8.83	645032	-5.16
**50920**	-3.05	158130	-7.13	311463	-8.73	645548	-8.72
**54235**	-5.04	161364	-4.00	316070	-11.28	645674	-10.87
**54397**	-7.35	162378	-8.10	319420	-9.35	647575	-7.13
**54700**	-8.43	163591	-8.07	319918	-5.66	649829	-7.72
**58924**	-4.22	164052	-7.08	320301	-2.45	650064	-8.13
**60847**	-4.01	164132	-9.46	322038	-5.25	650834	-7.93
**60855**	-6.04	164537	-8.15	323960	-2.36	652204	-9.10
**60933**	-11.90	166198	-6.60	329103	-7.93	652940	-4.16
**61811**	-2.87	166781	-8.04	329291	-6.22	653947	-4.61
**62543**	-6.73	168574	-4.78	331020	-3.27	654629	-9.94

**66370**	-2.62	169158	-7.59	333476	-10.23	654955	-7.16
**69574**	-2.05	170950	-5.70	333744	-8.94	655756	-6.58
**70127**	-5.49	171170	-2.35	333750	-8.84	656909	-5.88
**78522**	-7.89	174043	-7.51	338398	-6.69	658289	-6.49
**78878**	-2.55	174941	-7.89	338481	-8.04	658724	-2.58
**79594**	-9.59	179723	-6.52	338766	-8.42	659848	-8.48
**81459**	-5.81	180666	-2.97	339623	-10.21	659929	-2.46
**81832**	-2.76	180705	-8.17	342051	-7.62	671409	-1.92
**82444**	-1.34	181934	-6.58	343493	-5.11	672969	-5.40
**83536**	-7.09	184763	-6.09	344021	-3.82	673133	-9.83
**84086**	-6.32	202113	-6.22	346852	-5.70	676316	-8.93
**85530**	-9.46	202799	-6.79	351366	-6.29	676960	-0.53
**86538**	-7.58	203920	-8.18	358301	-3.93	677456	-8.46
**90596**	-3.14	204278	-7.67	359471	-6.14	677477	-9.87
**90610**	-2.66	204623	-8.21	366600	-7.43	677542	-2.18
**91690**	-7.31	205515	-10.88	367308	-4.60	680304	-8.40
**91999**	-9.26	205608	-7.73	371156	-2.34	685476	-10.01
**93327**	-7.61	208399	-8.89	380522	-8.88	693118	-5.29
**94749**	-4.31	209005	-4.13	400283	-5.23	693996	-7.00
**102345**	-7.03	210472	-8.45	401264	-6.32	694167	-6.93
**103877**	-1.37	211995	-7.88	402209	-5.52	695179	-6.22
**105793**	-9.86	216285	-8.03	402672	-4.96	697142	-6.68
**106137**	-10.34	216342	-8.27	403448	-5.20	697174	-10.56
**106151**	-8.61	216639	-8.26	403574	-8.37	697412	-5.20
**107691**	-6.82	216768	-8.82	404384	-7.16	697885	-5.68
**108869**	-7.46	216958	-8.34	507547	-8.91	697936	-3.18
**109830**	-5.88	216974	-8.21	508816	-7.76	700418	-4.09
**112133**	-4.49	220130	-9.53	509389	-7.80	701633	-6.21
**112367**	-7.44						

Table S5: The ligand-protein interactions between the PD-1/PD-L1 complex inhibitors and the PD-L1 protein of 5N2F.

Title	IC50 (nM)	Chain C	Chain D
**BMS-1001(1, 5NIU)**	2.25	10	Tyr56, Thr20, Ala18, Asp122, Lys124
**BMS-200 (2, 5N2F)**	80		Tyr56, Ala18, Phe19, Asp122
**BMS-3029 (3)**	2350		Tyr56, Thr20, Asp122, Lys124
**BMS-1166 (4, 5NIX)**	1.4		Tyr56, Thr20, Ala18, Asp122
**BMS-114 (5)**	43		Tyr56, Thr20, Ala18, Asp122
**BMS-1197 (6)**	1.85		Tyr56, Phe19, Thr20, Asp122, Lys124
**BMS-1205 (7)**	2.71		Tyr56, Thr20, Ala18, Asp122, Lys124
**BMS-1220 (8)**	6.07		Tyr56, Thr20, Ala18, Asp122, Lys124
**BMS-2002 (9)**	10	Asn63	Tyr56, Phe19, Thr20, Ala121, Asp122, Lys124
**BMS-1250 (10)**	1.19	Asn63	Tyr56, Ala18, Phe19, Thr20, Asp122
**BMS-1305 (11)**	0.92		Tyr56, Ala18, Phe19, Asp122
**BMS-1239 (12)**	148.9		Tyr56, Thr20, Ala18, Asp122, Lys124
**BMS-2010 (13)**	50		Tyr56, Thr20, Ala18, Asp122, Lys124
**BMS-3024 (14)**	5.54		Tyr56, Thr20, Asp122, Lys124
**BMS-16 (15)**	1945		Tyr56, Thr20, Asp122, Lys124
**BMS-82 (16)**	3186		Tyr56, Asp122, Lys124
**BMS-39 (17)**	4184	Gln66	Tyr56
**BMS-172 (18)**	107		Tyr56, Ala18, Asp122
**BMS-163 (19)**	93	Tyr56	Tyr56, Gly119, Ala121, Asp122, Tyr123
**BMS-202 (20, 5J89)**	18	Tyr56	Ala121, Asp122
**BMS-1043 (21)**	239.2	Asn63	Tyr56, Thr20, Ala18, Asp122
**BMS-8 (22, 5J8O)**	146		Tyr56, Asp122, Lys124
**BMS-107 (23)**	329		Tyr56, Thr20, Ala18, Asp12
**BMS-101 (24)**	1076		Tyr56, Asp122
**BMS-1016 (25)**	4.55		Tyr56, Phe19, Thr20, Asp122, Lys124
**BMS-1057 (26)**	985.8	Asn63	Tyr56, Thr20, Ala18, Asp122
**BMS-1095 (27)**	81.25	Asn63	Tyr56, Thr20, Ala18, Asp122
**BMS-1108 (28)**	624.2		Tyr56, Thr20, Ala18, Asp122
**BMS-1082 (29)**	828.4		Tyr56, Thr20, Ala18, Asp122

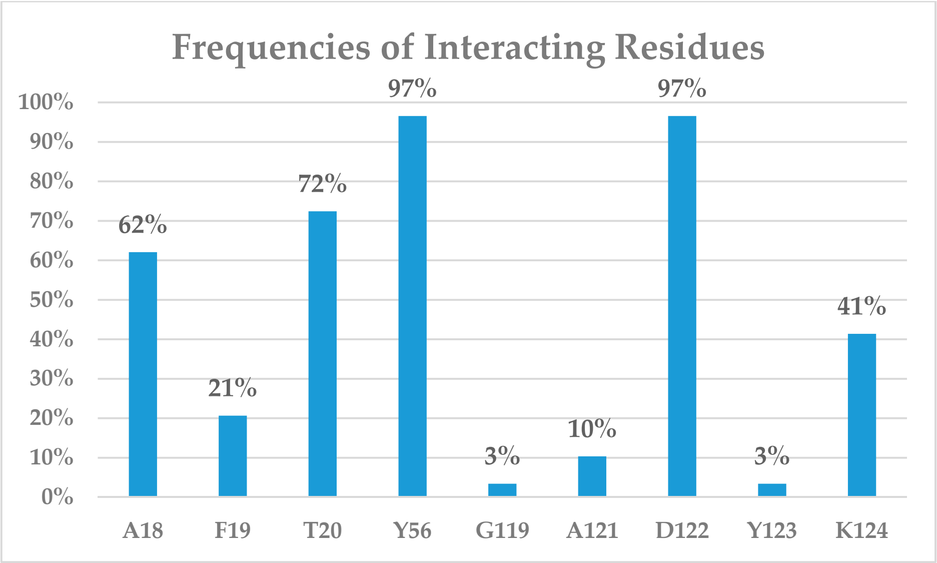

Figure S2: Interacting residues of PD-L1 with all 29 different inhibitors in the 5N2F model.

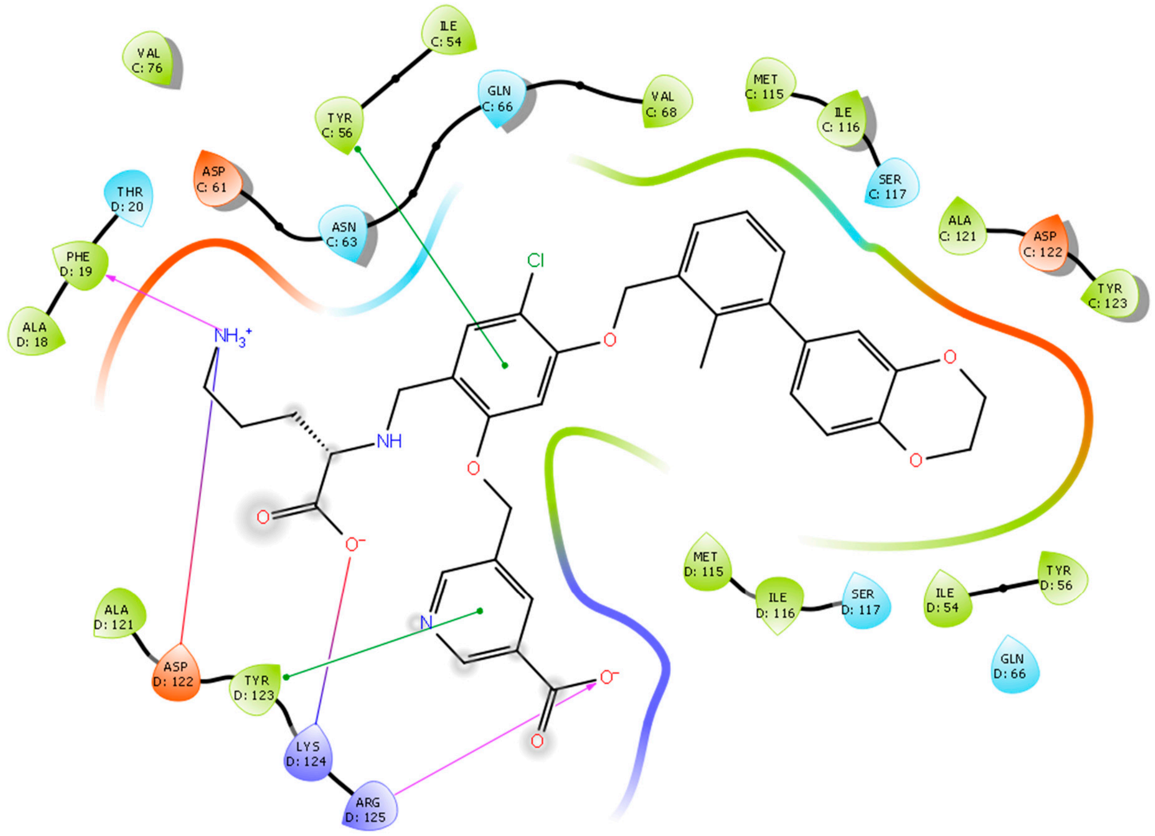

Figure S3: Protein-ligand interactions between 5NIU and BMS-2002 (**9**). It shows that main-chain carbonyl group of Phe19 and the side chain carboxylate of Asp122 form bonds with the amino moiety of ligand and that the Lys124 and Arg125 interact with two carboxylate groups of compound **9**. Tyr56 from chains C and D each forms π-π interactions with two different aromatic rings of **9**.
